# Sodium dodecyl sulfate‐coated silver nanoparticles accelerate antimicrobial potentials by targeting amphiphilic membranes

**DOI:** 10.1002/mlf2.12143

**Published:** 2024-12-03

**Authors:** Xiuyan Jin, Na Peng, Aoran Cui, Yue Liu, Xianqi Peng, Linlin Huang, Abdelaziz Ed‐Dra, Fang He, Yan Li, Shikuan Yang, Min Yue

**Affiliations:** ^1^ Department of Veterinary Medicine Zhejiang University College of Animal Science Hangzhou China; ^2^ School of Materials Science and Engineering, Institute for Composites Science Innovation Zhejiang University Hangzhou China; ^3^ Laboratory of Engineering and Applied Technologies, Higher School of Technology M'ghila University Campus, Sultan Moulay Slimane University Beni Mellal Morocco; ^4^ Laboratory of Animal Virology of Ministry of Agriculture Zhejiang University Hangzhou China; ^5^ Hainan Institute Zhejiang University Sanya China; ^6^ State Key Laboratory for Diagnosis and Treatment of Infectious Diseases, National Clinical Research Center for Infectious Diseases, National Medical Center for Infectious Diseases, The First Affiliated Hospital, College of Medicine Zhejiang University Hangzhou China; ^7^ Key Laboratory of Systems Health Science of Zhejiang Province, School of Life Science, Hangzhou Institute for Advanced Study University of Chinese Academy of Sciences Hangzhou China

**Keywords:** amphiphilic properties, antimicrobial agents, antimicrobial resistance, feed additive, silver nanoparticles

## Abstract

Compelling concerns about antimicrobial resistance and the emergence of multidrug‐resistant pathogens call for novel strategies to address these challenges. Nanoparticles show promising antimicrobial activities; however, their actions are hindered primarily by the bacterial hydrophilic–hydrophobic barrier. To overcome this, we developed a method of electrochemically anchoring sodium dodecyl sulfate (SDS) coatings onto silver nanoparticles (AgNPs), resulting in improved antimicrobial potency. We then investigated the antimicrobial mechanisms and developed therapeutic applications. We demonstrated SDS‐coated AgNPs with anomalous dispersive properties capable of dispersing in both polar and nonpolar solvents and, further, detected significantly higher bacteriostatic and bactericidal effects compared to silver ions (Ag^+^). Cellular assays suggested multipotent disruptions targeting the bacterial membrane, evidenced by increasing lactate dehydrogenase, protein and sugar leakage, and consistent with results from the transcriptomic analysis. Notably, the amphiphilic characteristics of the AgNPs maintained robust antibacterial activities for a year at various temperatures, indicating long‐term efficacy as a potential disinfectant. In a murine model, the AgNPs showed considerable biocompatibility and could alleviate fatal *Salmonella* infections. Collectively, by gaining amphiphilic properties from SDS, we offer novel AgNPs against bacterial infections combined with long‐term and cost‐effective strategies.

## Impact statement

Our study showed that sodium dodecyl sulfate (SDS)‐coated silver nanoparticles (AgNPs) have anomalous dispersive properties, which possess excellent antibacterial properties, compared to Ag^+^ ions (AgNO_3_). We compared the antibacterial actions of the AgNPs and Ag^+^ ions, likely due to their amphiphilic features, showing that the AgNPs efficiently penetrated the bacterial cell membrane, leading to the exudation of cellular components. We also observed differences in intercellular actions between the AgNPs and AgNO_3_ groups. Furthermore, AgNPs could safely mitigate *Salmonella* in vitro and in vivo infection models. Therefore, AgNPs may be used as potent antibacterial agents in animal feed to alleviate bacterial infections.

## INTRODUCTION

The emergence of antimicrobial‐resistant bacteria represents a significant threat to public and global health^1^, ^2^, ^3^, ^4^, ^5^, ^6^. The burden of antimicrobial resistance (AMR) is substantial, and AMR bacterial infections are becoming more challenging to treat therapeutically, leading to a higher risk of clinical complications and death^7^, ^8^, ^9^, ^10^, ^11^, ^12^, ^13^. According to the World Health Organization, AMR is one of the top 10 global public health threats facing human society, with an estimated 700,000 deaths annually attributed to drug‐resistant bacterial infections^8^, ^10^. Hence, there is an urgent and increasing need to develop more efficient and alternative antimicrobial agents^14^, ^15^.

Silver nanoparticles (AgNPs) have emerged as attractive alternatives to conventional antimicrobials, thanks to their effective properties and broad mechanisms of action^16^. Typically, silver (Ag) exerts antibacterial activities mainly in two layers: the first is extracellular action, where silver adheres to and disrupts the cell wall/membrane^17^, ^18^, and the second is intracellular action, where mutilation of intracellular biostructures (ribosomes, mitochondria, and vacuoles) and biomolecules (DNA, lipids, and proteins)^19^, extensive oxidative stress^20^, ^21^, and disruption of signal transduction pathways^22^ are involved. Compared to traditional antibiotics and other antimicrobial agents, AgNPs have several advantages, including their broad‐spectrum activity, in contrast to many conventional antibiotics, which are often effective only against specific types of bacteria^23^, ^24^. Nevertheless, traditional AgNPs have frequently been hindered by the amphiphilic properties of the bacterial membrane, which limits their effectiveness and wide application in both the veterinary and medical sectors.

Using a more adaptable but massive fabrication electrochemical approach, we established a novel pathway to break the AgNP's dispersible limit via an anchoring orientation with a tailorable surface amphiphilic ligand, sodium dodecyl sulfate (SDS)^25^. Compared with traditional AgNPs, which may lead to the development of bacterial resistance^26^, ^27^, our recently developed novel SDS‐coated AgNPs could overcome such limitations by resisting bacterial species‐induced aggregation^25^. Notably, the surface ligands helped the AgNPs penetrate the bacterial membrane, supporting their great potential for practical antibacterial applications. Nonetheless, the modes of bacteria‐killing action and the potential therapeutic applications of these AgNPs remain unaddressed. In this study, we examined multiple layers of the bacteriostatic effects and, more importantly, provided mechanistic insights, exploring the potential of nanosilver for biomedical applications.

## RESULTS AND DISCUSSION

### Preparation and characterization of AgNPs

**Figure 1:**
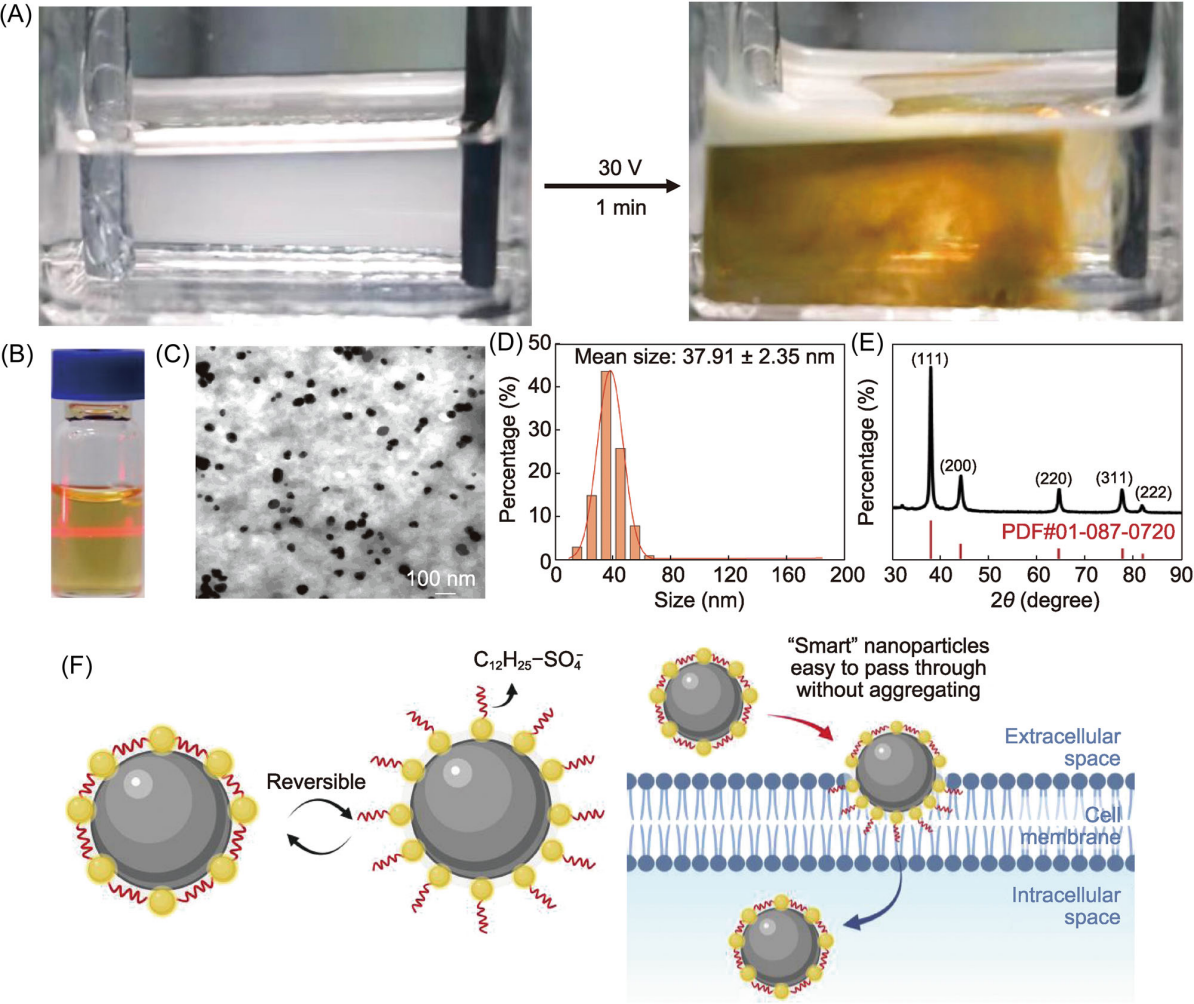
Sustained electrochemical preparation of silver nanoparticles (AgNPs) in electrolyte solution. (A) Production of AgNPs. AgNPs were obtained by electrodeposition in an electrolyte solution of silver nitrate (AgNO_3_) and sodium dodecyl sulfate (SDS). The AgNPs prepared here were continuously emitted from the electrode interface into the electrolyte solution, resulting in a yellow colloidal solution that formed within 1 min. (B) AgNPs showing the Tyndall effect when dispersed in water and indicating the homogeneity of the synthetic colloidal solution system. (C) Transmission electron microscope analysis of the AgNPs. (D) Particle size distribution of the AgNPs. (E) X‐ray diffraction analysis of the AgNPs. (F) Proposed mechanism for adapting ligands. The reversible orientation change of the surface ligands allowed them to adapt to surrounding liquids and easily pass through the bacterial membrane.

The preparation method of AgNPs has been described in detail in the literature^25^, ^28^. In this study, we adopted a similar preparation method and conducted a series of experiments for characterization (Figure 1A–E). The smart AgNPs were obtained by electrodeposition in an electrolyte solution of silver nitrate (AgNO_3_) and SDS. Dodecyl sulfate ions were anchored on the AgNPs during the electrodeposition process and could rotate to adapt to the surrounding liquid environment, breaking the nanoparticle's dispersible limit^25^. AgNPs can avoid aggregation even when exposed to bacteria‐secreted proteins, and the surface ligands can help the AgNPs penetrate the bacterial membrane, significantly enhancing their antibacterial performance (Figure 1F).^25^

### Potent antibacterial activity of AgNPs

**Figure 2:**
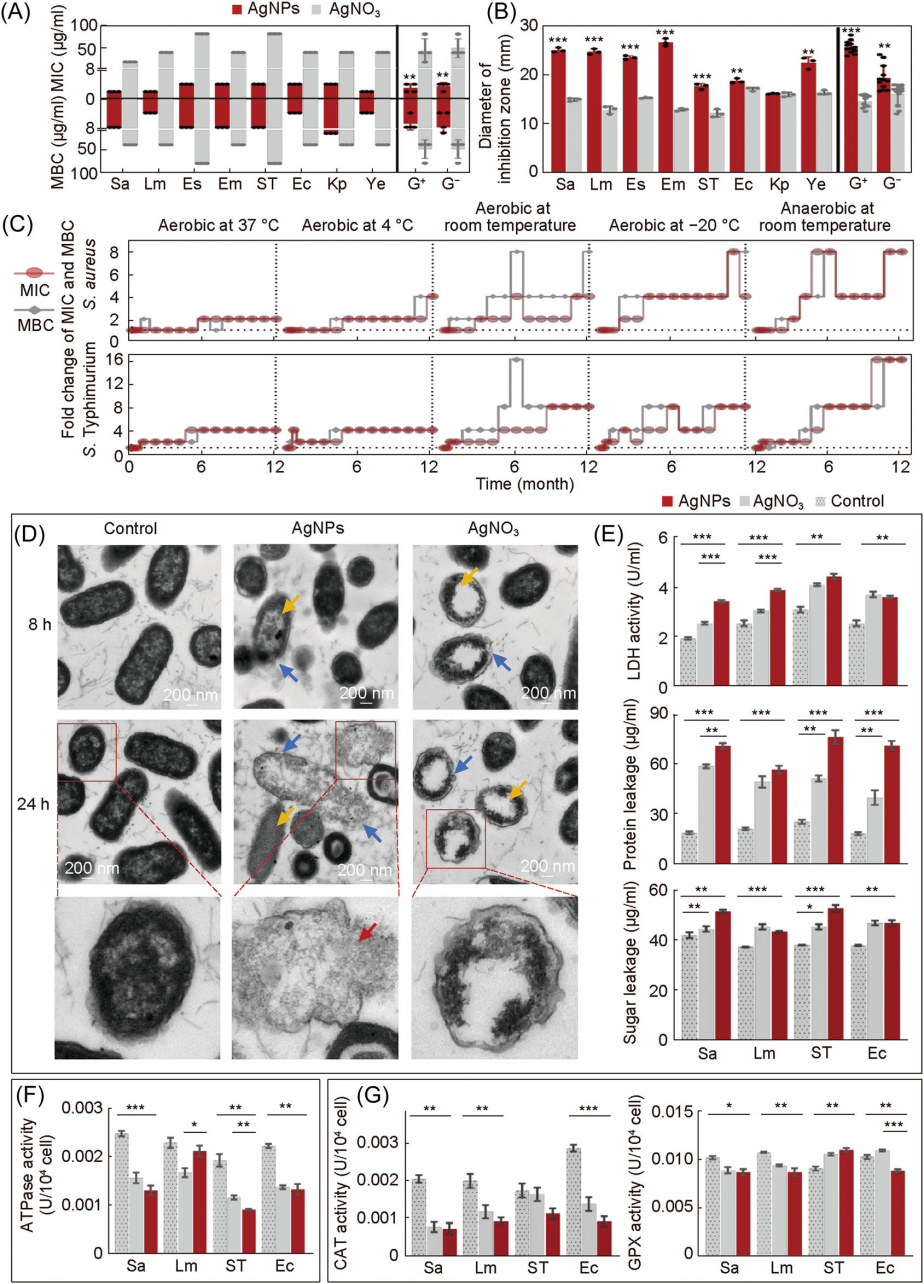
Bactericidal effect of AgNPs in vitro. (A) Minimum inhibitory concentration (MIC) and minimum bactericidal concentration (MBC) of AgNPs and originated derivative AgNO_3_. (B) Agar well diffusion assay for bacterial inhibition. (C) Long‐term bacterial inhibition and bactericidal efficacy of the AgNPs. The antibacterial effect of the AgNPs against *S. aureus* and *S*. Typhimurium under five different preservation conditions (aerobic at 37°C, aerobic at 4°C, aerobic at room temperature, aerobic at −20°C, and anaerobic at room temperature) at different time intervals (0, 0.25, 0.5, 1, 2, 3, 4, 5, 6, 7, 8, 9, 10, 11, and 12 months). (D) Visualization of AgNPs and AgNO_3_‐treated *S*. Typhimurium at 8 and 24 h using transmission electron microscopy analysis. The absence of AgNPs or AgNO_3_ was used as a control. Scale bars: 200 nm. (E) Contents of lactate dehydrogenase (LDH) and the efflux (protein and sugar) of bacterial contents after exposure to AgNPs and AgNO_3_. (F) ATPase activities after exposure to AgNPs and AgNO_3_. (G) Bacterial antioxidant enzyme activities after exposure to AgNPs and AgNO_3_, including glutathione peroxidase (GPX) and catalase (CAT). Red: bacteria treated with AgNPs. Gray: bacteria treated with AgNO_3_. Gray with points: control. Data were from at least three independent assays: *p < 0.05; **p < 0.01; ***p < 0.001. Sa, *Staphylococcus aureus*; Lm: *Listeria monocytogenes*; Es, *Enterococcus faecalis*; Em, *Enterococcus faecium*; ST, *S*. Typhimurium; Ec, *Escherichia coli*; Kp, *Klebsiella pneumoniae*; Ye, *Yersinia enterocolitica*. *S. aureus*, *L. monocytogenes*, *E. faecalis*, and *E. faecium* are Gam‐positive (G^+^) bacteria. *S*. Typhimurium, *E. coli*, *K. pneumoniae*, and *Y. enterocolitica* are Gram‐negative (G^–^) bacteria.

The antibacterial activity of the AgNPs and AgNO_3_ was evaluated using bacteria‐killing and inhibition assays^29^, ^30^ against eight representative pathogenic bacterial strains, including four Gram‐negative and four Gram‐positive bacteria, which posed a major threat to public health^31^, ^32^. The antibacterial efficiency of the AgNPs against bacteria was higher than that of AgNO_3_ (Figure 2). The inhibitory effect of the AgNPs on the Gram‐positive bacteria was more potent than their impact on the Gram‐negative bacteria (Figure 2A). Gram‐positive bacteria have multilayered cell walls of peptidoglycan^33^, ^34^. Likely due to differences in the structure, thickness, and negative charge of the cell wall, silver‐containing antibacterial agents have better effects on Gram‐negative bacteria^35^. It was assumed that the structural similarities between the surface ligands of the AgNPs and the bacterial membranes^25^ enabled the AgNPs to show a powerful antibacterial effect when facing bacteria with more complex cell wall structures. Minimum inhibitory concentration (MIC) and minimum bactericidal concentration (MBC) assays were used to evaluate the antibacterial activity of the SDS solution (Figure S1). The results demonstrated that SDS had minimal antibacterial capability when used alone; its antibacterial capability was only achieved when combined with the AgNPs. Furthermore, an agar well diffusion assay was used to evaluate the antibacterial activity of the AgNPs and AgNO_3_ (Figure 2B). Biofilm formation was observed under aerobic conditions at 37°C and 22°C, and the inhibition rate of the AgNPs on bacterial biofilm formation was generally higher than that of AgNO_3_ (Figure S2A).

The antibacterial effect of AgNPs was also investigated under various conditions over different time periods as one of the most critical parameters of the prerequisites for using as biomaterials. The MIC and MBC of the AgNPs were measured under five different conditions on two strains of *Staphylococcus aureus* and *S*. Typhimurium separately (Figure 2C). The effects of storage conditions on the bacteriostatic effect of the AgNPs over time were examined. The results showed that under storage conditions of 37°C and 4°C, the AgNPs maintained the best antibacterial activity, with a limited difference during the examined period. Under the conditions of −20°C and room temperature, the antibacterial activity of AgNPs changed dramatically, indicating that these conditions were not conducive to maintaining the original structure of the AgNPs and that low temperatures may impact the stability of the AgNP colloidal solution. These results also indicate that temperature is the main parameter affecting the antibacterial activities of AgNPs and that a stable temperature is essential to conserving their antimicrobial activities.

### Bactericidal mechanisms of AgNPs

In a time‐dependent killing curve analysis (Figure S2B), we explored the appropriate antibacterial concentration and used this concentration to provide a reference for the subsequent experiments.

To examine the action on the bacterial membranes, the activities of the AgNPs and AgNO_3_‐disrupting bacterial membranes were directly examined under transmission electron microscopy (TEM). The results showed that the bacterial membrane was completely obliterated after exposure to the AgNPs and AgNO_3_ (Figure 2D) compared to the control. The interaction of *S*. Typhimurium with AgNPs/AgNO_3_ resulted in the thinning or even disappearance of the cell walls, membrane deformation or crumpling, membrane blebbing and contents release (indicated by the blue arrow), and cytoplasmic disorders in some bacteria (indicated by the yellow arrow). Unlike AgNO_3_, AgNPs led to the complete rupture of bacterial cells. When the cells were aggregates composed of many dense granules and cytoplasm, nanoparticles were condensed in the cell membrane and wall as clusters and were seen inside the cell in some regions (indicated by the red arrow). Significant changes in the cell morphology of bacteria have also been observed in several studies with results that align with our observations^36^, ^37^, ^38^.

Lactate dehydrogenase (LDH) is rapidly released into the cell culture supernatant when the membrane is damaged and is a key feature of cellular damage^39^, ^40^. The LDH activity in the bacterial supernatant after AgNP treatment was higher than that of AgNO_3_ treatment (*p* < 0.001) on *S. aureus* and *Listeria monocytogenes* (Figure 2E), which indicated that the AgNPs were more efficient for cellular damage than AgNO_3_, which is consistent with a previous study^41^. The results suggested that the permeability of bacterial cell membranes might be altered after AgNP intervention. The protein and reducing sugar content assays were performed to verify the damage to the bacterial cell membrane. The release of cellular content, such as proteins and sugars, is a reliable indicator of cell membrane permeability and damage^42^. Protein leakage and reducing sugar concentrations significantly increased after co‐culture with the AgNPs compared with the control group (Figure 2E), but there were no significant differences in the reducing sugar concentrations in *L. monocytogenes* and *Escherichia coli* supernatants under AgNO_3_ treatment. This indicated that AgNPs led to a more severe leakage of bacterial cell contents, which is consistent with the conclusion of Kim^43^. Altogether, AgNPs resulted in more severe membrane damage than AgNO_3_.

In the aforementioned investigation, we found damage inflicted by AgNPs on bacterial membranes both externally and internally through direct observation and detection of the bacterial contents. The bacterial cell membrane, serving as the protective shell for bacteria, holds significant physiological significance^44^ for the bacterial cell envelope. Contact with AgNPs initiates aggressive assaults on bacterial membranes through the AgNPs’ amphiphilic characteristics, marking the initial step toward the defeat of the bacteria.

To investigate the role of metabolic action, the activity and quantity of ATPases in the bacteria treated with AgNPs or AgNO_3_ were examined. It was found that the activity and quantity were significantly decreased (Figure 2F) because AgNPs or AgNO_3_ could lead to bacterial ATPase degradation and dysfunction by directly interacting with bacterial ATPases or enhancing bacterial oxidative stress^45^. Compared to AgNO_3_, AgNPs could, directly and indirectly, disrupt intracellular ATPase activity while disrupting bacterial cell membrane likely due to their superior specific surface area and surface chemical properties^46^. Overall, the two silver solutions could interfere with bacterial metabolism because ATPase activity was significantly affected.

The nanoparticle‐mediated generation of reactive oxygen species (ROS) has been shown to disrupt the electron transport components of the cell membrane and regulate various antioxidant enzymes, including nicotinamide adenine dinucleotide phosphate (NADPH)‐dependent flavones, catalase (CAT), and glutathione peroxidase (GPX). ROS production causes oxidative stress and damages cellular components^47^, ^48^, ^49^, ^50^, ^51^. Reduction activities in the CAT and GPX antioxidant enzymes in bacteria may disrupt the dynamic balance between the production and elimination of ROS in bacteria, leading to a series of related reactions, such as cell membrane damage, weakened DNA replication, and ATP dysfunction, which result in impaired bacterial metabolism^48^. To further examine the oxidative damage of cells induced by AgNPs or AgNO_3_, we evaluated the activity of oxidative stress reaction‐relevant enzymes, including CAT and GPX. Compared to the control, the AgNPs mainly inhibited the CAT activity of *E. coli* compared to the other tested bacteria (*p* < 0.001; Figure 2G). However, except for *S*. Typhimurium, all the bacteria showed decreased GPX activity under AgNPs and AgNO_3_ stress (Figure 2G). GPX activity in *S. aureus* and *L. monocytogenes* was significantly reduced under both treatments and increased in AgNPs‐treated *S*. Typhimurium and AgNO_3_‐treated *E. coli* and *S*. Typhimurium.

The aforementioned results indicated that after AgNPs disrupt the cell membrane, they encounter substances inside the membrane, including ATP and antioxidant enzymes vital for bacterial survival. This interaction affects bacterial metabolism and oxidation, ultimately leading to bacterial death. As critical survival factors, ATP enzymes are among the important targets for drug therapy. Our findings support the application of targeted therapy in combating bacteria^52^, ^53^.

### The effect of AgNPs on the regulation of gene expression in *S*. Typhimurium

**Figure 3:**
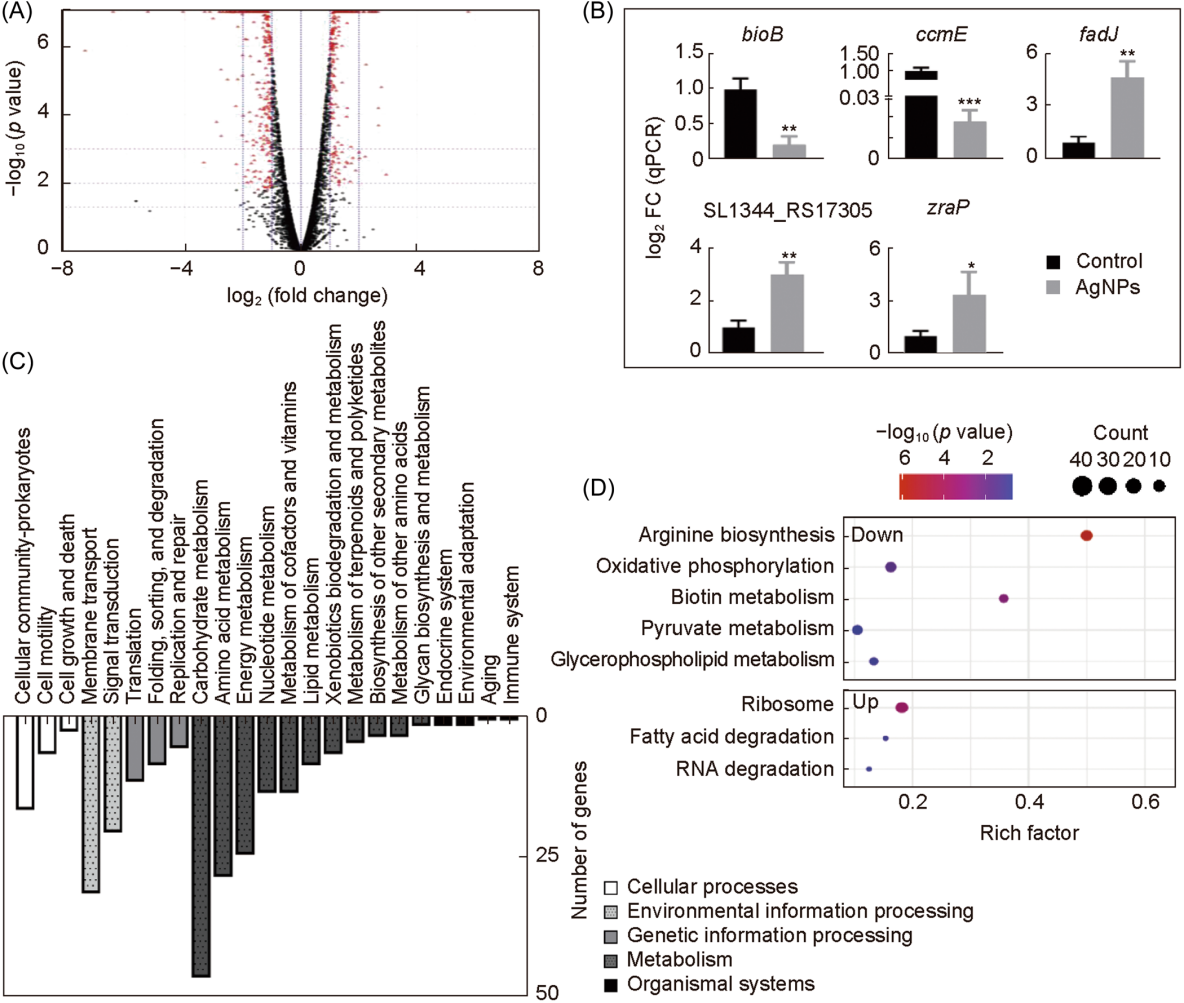
Effect of AgNPs on *S*. Typhimurium gene expression. (A) Volcanic map of differentially expressed genes (DEGs). DEGs: red point; upregulated genes: *X*‐axis > 0; downregulated genes: *X*‐axis < 0. (B) mRNA levels of the DEGs by quantitative reverse transcription polymerase chain reaction (RT‐qPCR). Each scatter represents the expression level of each gene treated with AgNPs. The relative expression level of the target genes was measured with the 2^–ΔΔCt^ method, and 16S rRNA was used as the reference gene. All tests were performed at least three times. (C) Kyoto Encyclopedia of Genes and Genomes (KEGG) pathway classification of the DEGs. (D) KEGG pathway group enrichment of the DEGs. *p < 0.05; **p < 0.01; ***p < 0.001.

To further understand the molecular details underlying the phenotypes, the overall genes at the transcription level in *S*. Typhimurium under AgNPs were investigated by RNA‐seq. A total of 580 differentially expressed genes (DEGs) were detected in the AgNP‐treated group versus the control group (Figure 3A). The RT‐qPCR results were consistent with RNA‐seq results (Figure 3B). A pathway enrichment analysis utilizing the Kyoto Encyclopedia of Genes and Genomes (KEGG) database was conducted to elucidate the metabolic and signal transduction pathways associated with the DEGs (Figure 3C).

In addition, a scatter plot of the KEGG enrichment results was generated (Figure 3D). The main enriched pathways of *S*. Typhimurium cells under AgNP stress were illustrated (Table S1), including glycerophospholipid metabolism, fatty acid degradation, arginine biosynthesis, oxidative phosphorylation, RNA degradation, biotin metabolism, pyruvate metabolism, and ribosome. Glycerophospholipids are vital components of the double‐membrane envelope of Gram‐negative bacteria, and the homeostasis of membrane glycerophospholipids is closely related to bacterial stress responses and adaptive mechanisms^53^, ^54^. The genes involved in the glycerophospholipid metabolism revealed that four DEGs were significantly downregulated after treated by the AgNPs, suggesting that cells could modulate membrane fluidity to enhance membrane stability when exposed to challenging conditions^55^. In bacteria, fatty acids are a significant component of phospholipids, and changes in phospholipids could affect cell membrane structure and function^56^. The *fadJ* and *fadI* genes were significantly upregulated in *S*. Typhimurium treated with AgNPs, suggesting that AgNPs can activate fatty acid degradation (Table S1). Hence, the AgNP treatment inhibited bacterial glycerophospholipid metabolism and triggered fatty acid degradation, overcoming the adaptive response to external disturbances by destroying cell membrane integrity and membrane stability.

Many previous studies have shown that nanoparticles have oxidase‐like activity, which can cause severe oxidative damage to bacteria to achieve sterilization^20^, ^57^, ^58^. Some pathways identified in the KEGG enrichment results treated with AgNPs have been closely linked to oxidative stress responses, such as arginine biosynthesis, oxidative phosphorylation, and RNA degradation. Others have been closely linked to metabolic disturbances, such as biotin metabolism, oxidative phosphorylation, pyruvate metabolism, and the ribosome (Table S1).

### In vitro toxicity and protective capacity of AgNPs against *Salmonella* infections

**Figure 4:**
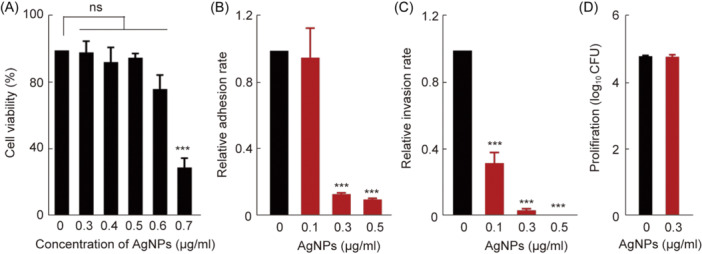
In vitro toxicity and protective capacity of AgNPs against *Salmonella* infections. (A) In vitro biocompatibility effect of AgNPs on the viability of the murine RAW264.7 macrophage cell detected using the CC‐K8 assay. (B–D) The dose effects of AgNPs in bacteria and cell interactions. For *S*. Typhimurium in the RAW264.7 cells, the geometric means of CFUs per cell were obtained from three independent experiments for cell adhesion, invasion, and proliferation. ***p < 0.001; ns, no significance.

Biological safety issue is essential for transforming antimicrobial materials from basic research to clinical applications. In this investigation, the cytotoxic effect of AgNPs was evaluated on RAW264.7 cells using the CCK‐8 assay to select a safe concentration. It was found that AgNps were safe for cells in vitro at concentrations below 0.6 μg/ml. (Figure 4A). The adhesiveness, invasiveness, and intracellular proliferation of *S*. Typhimurium were determined by measuring the CFUs. The relative adhesion and invasion rates in the AgNP‐treated group were slightly lower than those in the *Salmonella*‐infected group (*p* < 0.001) (Figure 4B,C), and the AgNPs were associated with decreased adhesion and invasion. We also examined proliferation using a gentamicin protection assay. At 24 h post infection, the RAW264.7 cells were lysed, and the CFUs were enumerated, but there was no significant difference between the *Salmonella*‐infected group and the AgNP‐treated group (Figure 4D). *Salmonella* utilized macrophages as vectors for systemic dissemination throughout the host^59^, and the reduction of relative adhesion and invasion rates indicated that AgNPs exerted direct action on the bacteria itself to disrupt their infection within the host.

### In vivo protective efficacy of AgNPs against *Salmonella* infections

**Figure 5:**
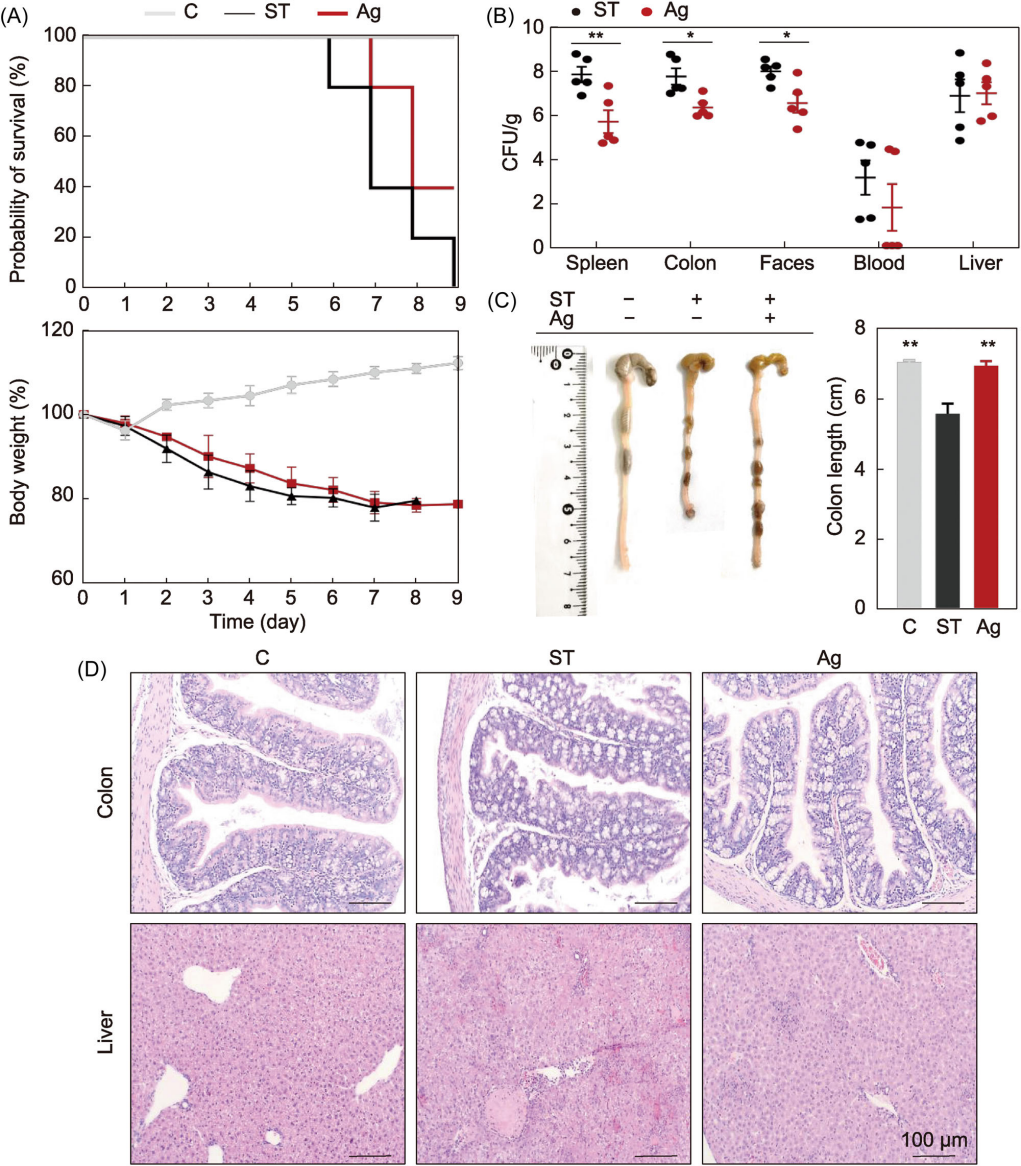
In vivo protective efficacy of AgNPs against *Salmonella* infections. (A) Comparative analysis of survival and body weight. Changes in the probability of survival and body weight over time after infection were observed from the day of infection. (B) Bacterial tissue load analysis. Amount of *Salmonella* was detected in the spleen, colon, feces, blood, and liver, but not detected in any control group sample (data not shown). (C) A representative colon length image and a summary of all examined colon length data. (D) Hematoxylin and eosin (H&E) staining analysis of liver and colon sections of each group from the representative samples. *p < 0.05; **p < 0.01. C, control group; ST, *Salmonella* infection group; Ag, *Salmonella* infection and AgNP‐treated group. Of 10 mice, five were used for clinical trials, and five were used to observe survival and growth. Samples were collected on Day 5 post infection.

*Salmonella* infection results in approximately 1.35 million cases of illness and 420 fatalities annually in the United States^60^. *Salmonella enterica* serovar Typhimurium is one of the most important serovars transmitted from animals to humans in most parts of the world^61^, ^62^. In the transcriptome results, we found gene changes related to *Salmonella* infection (Figures S3 and S4). This suggested that AgNPs had a potential role in preventing *Salmonella* infection. The main enriched pathways have been illustrated (Table S2). AgNPs as feed additives could offer a novel avenue for establishing antibacterial barriers against pathogenic bacterial infections, including *Salmonella*. The mortality of the mice in the *Salmonella* infection group was 100% on Day 9, which was higher than that observed in the AgNP‐treated group (40%) (Figure 5A). Weight loss in the AgNP‐treated group was slightly lower than that of the *Salmonella* infection group in the first 7 days, but there was no significant difference, suggesting a role for AgNPs as feed additives against *Salmonella* infections.

*Salmonella* overcomes intestinal barriers to interact with the intestinal epithelium and penetrates deeper into the tissues of the host^63^, ^64^, ^65^, ^66^, and long‐term infection could lead to sepsis and death^67^, ^68^. In this study, the decreased viable counts of *Salmonella* in tissue, feces, and blood on Day 5 post infection confirmed the success of the treatment with AgNPs. The amount of *Salmonella* recovered from the spleen, colon, and feces decreased significantly (*p* < 0.01, *p* < 0.05, and *p* < 0.05, respectively) in the AgNP‐treated group (Figure 5B), which was in line with the findings of previous studies^69^. These results indicated that AgNPs could significantly inhibit *Salmonella* invasion.

Colon shortening is a critical parameter for evaluating the severity of enteritis^70^. Colon length recovered significantly in the AgNP group (*p* < 0.01; Figure 5C). The host intestine is the main site for the entry of pathogens, thereby causing systemic infection, and the liver is an essential target for *Salmonella*. To evaluate the tissue damage, the mice's livers and colons were dissected and prepared for paraffin sections and hematoxylin and eosin (H&E) staining (Figure 5D).^68^ In the group infected with *Salmonella*, the colonic mucosa exhibited signs of damage, characterized by necrotic enteritis, cellular vacuolation, the demise of enterocytes, and a widespread inflammatory cellular reaction (Figure 5D). In contrast, in the AgNP group, infiltration was significantly reduced. The intestinal mucosae were improved with an apparent reduction in mucosal and submucosal inflammatory aggregation. In the *Salmonella*‐infected mice, the livers showed congestion of the central and portal veins, focal aggregations of mononuclear inflammatory cells, and the appearance of inflammatory cells. In addition, the hepatocytes showed vacuolar degeneration, necrotic changes, and mononuclear cell infiltration between the hepatic cords, indicating the occurrence of acute hepatitis in the *Salmonella*‐infected mice. The group treated with AgNPs exhibited a subdued inflammatory response, significantly lower than that of the untreated group, and the hepatocytes’ integrity and structural composition were preserved. The alleviating effect was similar to that found in previous studies^69^. These results indicated that AgNPs could protect mice against *Salmonella* infection‐induced damage to internal organs.

Altogether, AgNPs directly exerted antibacterial properties and effectively alleviated enteritis and systemic infections caused by *Salmonella*. The excellent preventive and therapeutic effects of AgNPs indicated that they have the potential to become an effective feed additive for the prevention of intestinal pathogen infections.

**Figure 6:**
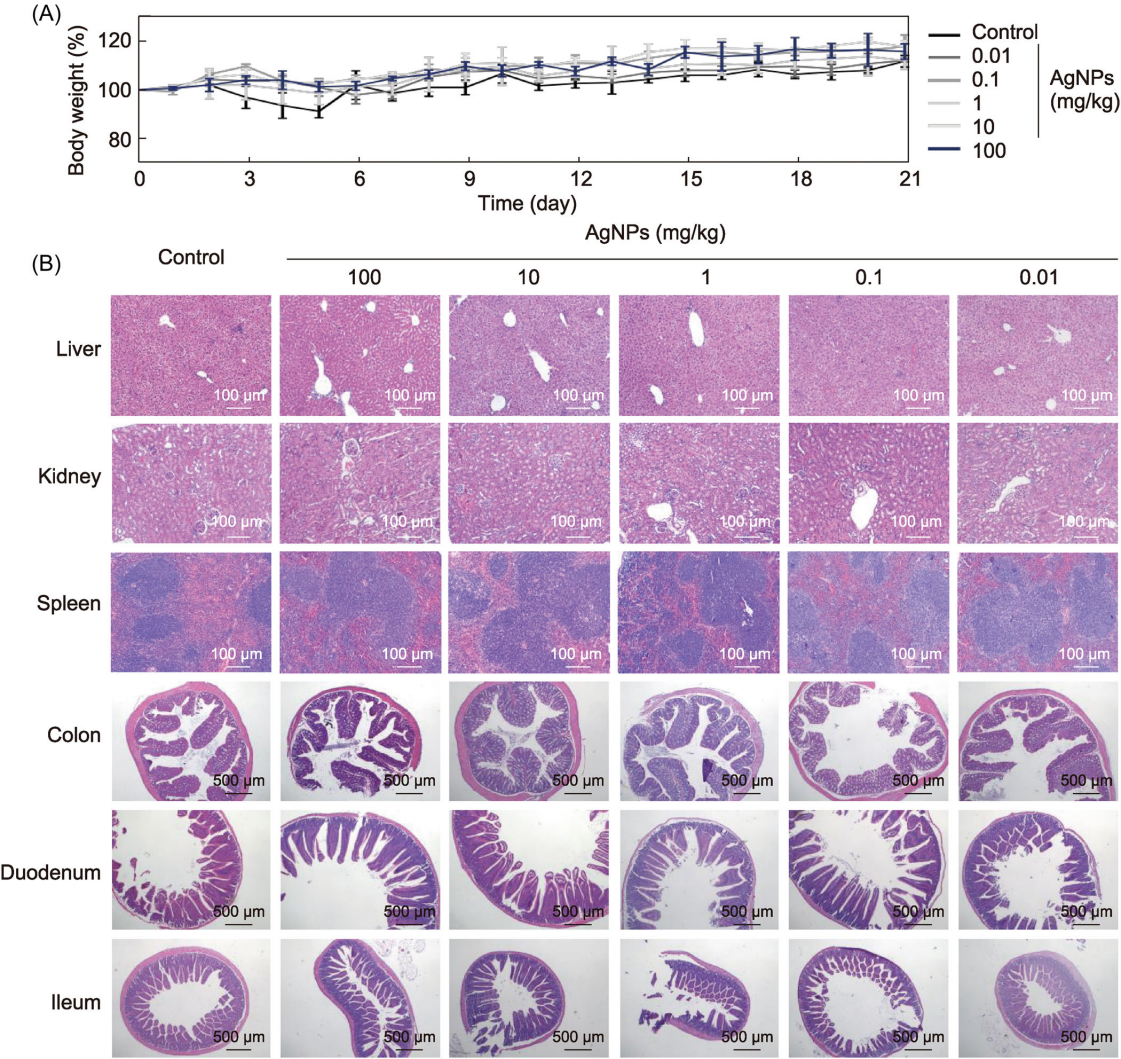
In vivo toxicity of AgNPs. (A) In vivo biocompatibility evaluation of the AgNPs by acute toxicity. AgNPs (0.01, 0.1, 1, 10, and 100 mg/kg) were given orally to mice once daily for 7 days. For the control groups, ddH_2_O was administered to the animals through the same route as in each test. Within 21 days of acute toxicity, body weight was measured. (B) Tissue pathology. Hematoxylin and eosin (H&E)‐stained liver, kidney, spleen, colon, duodenum, and ileum sections of each group were collected on Day 10 post infection.

In vivo acute toxicity was further assessed by administering different doses of AgNPs to mice for seven consecutive days. Strikingly, mice in the groups treated with AgNPs displayed gradual increase in their body weight (0.01–100 mg/kg), with no statistically significant difference from the controls (Figure 6A). No detected abnormalities were found in the H&E‐stained sections of the three major organs (liver, kidney, and spleen). Three types of intestinal tissues (colon, duodenum, and ileum) were taken from the mice in the treatment groups with different concentrations of AgNPs, and it could be seen that the sections from AgNP treatment groups were essentially consistent with the controls, with no visible tissue damage (Figure 6B). The aforementioned results suggested that AgNPs may show excellent safety after oral administration, which may be used for clinical investigations.

In summary, our study revealed that SDS‐coated AgNPs exhibited unusual dispersive properties and exceptional antibacterial efficacy. These AgNPs induced leakage of bacterial cellular contents and caused severe damage to the bacterial cell surface, as supported by transcriptomic analyses indicating cellular membrane damage. Furthermore, AgNPs affected bacterial metabolism and induced oxidative stress. Notably, they maintained stable antibacterial activity for over a year and demonstrated excellent biocompatibility, with no observed toxicity to animals while effectively combating *Salmonella* in vivo infections. Together, we offered novel AgNPs against bacterial infections combined with long‐term and cost‐effective strategies.

## MATERIALS AND METHODS

### Preparation of AgNPs

The AgNPs were synthesized following our previous methods^25^. To prepare the electrolytes, 50 ml of 30 mM AgNO_3_ and 90 mM of SDS solutions were used. Electrodeposition was carried out under the two‐electrode system at 30 V for 300 s. The cathode was an aluminum plate (2 cm × 2 cm), and the anode was a carbon rod (diameter: 0.5 cm). AgNPs continuously diffused from the cathode electrode surface to the electrolyte, forming a yellow mist. After electrodeposition for 5 min, the black precipitation was filtered, and the yellow AgNPs were used for further characterization and antibacterial experiments.

### Bacterial strains and reagents

*S. aureus* ATCC 25923, *L. monocytogenes* ATCC 13932, *S*. Typhimurium SL 1344, and *Yersinia enterocolitica* ATCC 23715 were obtained from the Molecular Microbiology and Food Safety Laboratory of Zhejiang University and used as the standard experimental strains of this study. Additionally, *Enterococcus faecalis*, *En. faecium*, *E. coli*, and *Klebsiella pneumoniae* were clinically isolated from the affiliated animal hospital of Zhejiang University and used to enhance the bacterial spectrum of this study. Luria‐Bertani (LB) medium, Mueller‐Hinton (MH) medium, Brain Heart Infusion (BHI), tryptone, and yeast extract were supplied from Oxoid (Biodee, Co.).

### Antibacterial activity of AgNPs

The MIC assay was used to evaluate the antibacterial activity of AgNPs or AgNO_3_ (composed of Ag^+^ ions as control) on eight species of bacteria using the broth dilution method according to the previously described protocol^71^. The experiments were carried out in 100 µl of AgNPs or AgNO_3_ with decreasing concentrations. The bacterial growth was evaluated by a microplate reader (TECAN Infinite® F50, Switzerland). *E. coli* ATCC 25922 was used as a quality control strain to validate antimicrobial susceptibility testing. Additionally, the MBC was determined by inoculating 10 µl of wells that didn't present bacterial growth on LB agar, and then the plates were incubated at 37°C for 18–24 h. The trials were conducted on three separate occasions to ensure the accuracy and reliability of the MIC and MBC values. In addition, the antimicrobial efficacy of both AgNPs and AgNO_3_ was evaluated using the Oxford agar diffusion assay^72^.

### Evaluation of parameters affecting the antibacterial activity of AgNPs

To determine the optimal antibacterial activity of the electrodeposited AgNPs, we measured the MIC and MBC of AgNPs under different storage conditions. The AgNPs prepared in the same batch were stored under five conditions: anaerobic at room temperature, room temperature (with dynamic change at the laboratory), 37°C, 4°C, and −20°C. The sample was divided into several parts to avoid repeated freezing and thawing of samples at 4°C and −20°C and stored in different conditions. The MIC and MBC of AgNPs were determined periodically (0, 0.25, 0.5, 1, 2, 3, 4, 5, 6, 7, 8, 9, 10, 11, and 12 months) against Gram‐positive bacteria (*S. aureus*) and Gram‐negative bacteria (*S*. Typhimurium). The experimental steps of MIC and MBC were the same as mentioned above.

### TEM analysis

A TEM analysis was carried out to investigate the effects of AgNPs or AgNO_3_ on bacterial cell morphology. Briefly, each bacterial cell was treated with AgNPs or AgNO_3_ at a concentration of 156.25 µg/ml (2 × MIC) and incubated for 8 and 24 h at 37°C on an orbital shaker at 180 rpm. The untreated cells were used as a control. Bacteria cells (*S*. Typhimurium) were collected by centrifugation at 5000 rpm for 10 min, added to a 2.5% glutaraldehyde solution, and placed at 4°C overnight. After rinsing, decolorizing, dehydrating, and embedding, the sample was sliced with the LEICA EM UC7 ultrathin slicer to a thickness of 70–90 nm. The slices were stained with a heavy metal salt^73^. After drying, the bacterial morphology was observed in the TEM (H‐7650, Hitachi‐Science & Technology).

### Evaluation of membrane integrity

To evaluate the bacterial cell membrane integrity, the contents of LDH, protein, and reducing sugar in the cytoplasm of cells were measured. Briefly, 3 × 10^9^ CFU/ml bacterial cells (*S. aureus*, *L. monocytogenes*, *S*. Typhimurium, and *E. coli*) were treated with 156.25 µg/ml (2 × MIC) AgNPs or AgNO_3_ for 24 h at 37°C. The supernatant was collected by centrifugation at 10,000*g* for 5 min at 4°C and stored at −20°C until subsequent use. The bacterial suspension cultured without AgNPs and AgNO_3_ was used as the control sample. According to the LDH assay kit (Sangon Biotech), the OD_450_ was determined in an alkaline solution to measure LDH. The modified Bradford method was used to determine the protein concentration using a protein assay kit (Sangon Biotech)^42^. The concentration of reduced sugar was determined by the 3,5‐dinitrosalicylic acid analysis method^74^ using the sugar detection kit (Sangon Biotech, China).

### Bacterial metabolic activity assay (ATPase)

The AgNPs and AgNO_3_ were added at a concentration of 156.25 µg/ml (2 × MIC) to bacteria in the logarithmic phase and cultured for 24 h at 22°C or 37°C, then the bacteria were collected by centrifugation at 5000 rpm at 4°C for 10 min. Untreated bacteria were used as a control. Afterward, the bacteria were submitted to an ultrasonic crusher by using the extraction solution to break the cells on ice. Then, the crushed bacteria were centrifuged at 8000 *g* at 4°C for 10 min, and the activity of ATPase was determined by using a commercial kit (Solar Science & Technology). The detailed protocol was documented previously.^75^

### In vitro determination of antioxidative stress

Bacterial cells (3 × 10^9^ CFU/ml) were treated with 156.25 µg/ml of AgNPs or AgNO_3_ for 24 h at 37°C. The supernatant was collected by centrifugation at 10,000*g* for 5 min at 4°C and stored at −20°C until subsequent use. To evaluate the effects of AgNPs and AgNO_3_ on bacterial antioxidant enzymes, including GPX and CAT, we centrifuged the solution at 5000 rpm at 4°C for 10 min and then added the extracting solution. We used the ultrasonic crusher to treat the bacteria on the ice. Afterward, the GPX and CAT activities in the sample were measured according to the instructions of the GPX and CAT assay kit (Solar Science & Technology).

### Transcriptome analysis

*S*. Typhimurium incubated with 78.125 µg/ml AgNPs for 24 h was used for transcriptome sequencing, whereas *S*. Typhimurium was treated with only LB medium as a control. The library preparation and sequencing were completed by the Meige Gene Company. Trimmomatic (v.0.36) was used for quality control and data filtering of raw data in FASTQ format. The quality‐controlled sequences were compared to those of ribosomal RNA (rRNA) in the NCBI Rfam database, and rRNA sequences were removed by Bowtie2 (v2.33). The remaining mRNA sequences were mapped to the reference genome by Hisat2 (2.1.0). For gene expression analysis, the FPKM for each gene was calculated. The edge R (v3.16.5) was used to represent DEGs between two conditions/groups, and the resulting *p‐*values were adjusted by Benjamini and Hochberg, controlling for the false discovery rate. Genes with FDR ≤ 0.05 and |log_2_‐fold change| ≥ 1 were used for subsequent enrichment analysis^76^. Enrichment analysis of DEGs by gene ontology (GO) and KEGG was performed with clusterProfiler (v3.4.4) and corrected for gene length bias. GO terms with FDR ≤0.05 and KEGG pathways are significantly enriched for DEGs and used for subsequent analysis^77^, ^78^, ^79^, ^80^. The raw data produced in this study are available at Bioproject (PRJNA931462).

### RT‐qPCR

Representative genes of relevant pathways were analyzed by RT‐qPCR to evaluate the validity of transcriptome sequencing results. Total RNA was reverse‐transcribed to cDNA using HiScript III RT SuperMix for qPCR (+gDNA wiper) (Novozymes). Primers were synthesized by Tsingke Bio. PCR reactions were then performed using the ChamQ Universal SYBR qPCR Master Mix (Novozymes) with the following cycling conditions: 95°C for 10 min, followed by 40 cycles of 95°C for 30 s and 60°C for 20 s. The relative expression levels of target genes were determined by the 2^–^^ΔΔCt^ method, and 16S rRNA was used as the internal reference gene. All experimental results were performed in three biological replicates. Primer designs are listed in Table S3.

### Bacterial adhesion, invasion, and proliferation assay for macrophages

RAW264.7 was seeded onto 96‐well plates at a density of 5 × 10^4^ per well to allow cells to attach to the bottom of the wells. Three separate plates for one set of phagocytic cell invasion assays were set up and marked with “adhesion,” “invasion,” and “proliferation,” respectively. Cytotoxic concentration was detected before *Salmonella* (*S*. Typhimurium) infection by using the CCK‐8 assay (in the last cell passage). 200 μl bacteria with 1 × 10^6^ CFU cells were added in each well with multiplicity of infection (MOI) of 20:1. AgNPs in non‐toxic concentrations were added to the adhesive and invasive plates when *Salmonella* cells were added (0.1, 0.3, and 0.5 µg/ml), whereas AgNPs were added to the proliferation plates after *Salmonella* infection (0.3 µg/ml). Then, the key steps were performed according to the previously described protocol to examine the adhesion, invasion, and proliferation of *Salmonella* in RAW264.7 macrophage cells.^81^

### Intervention of murine enteritis caused by *Salmonella*

All mice were fed adaptively for 1 week and then divided into three groups: healthy, *Salmonella* (*S*. Typhimurium) infection, and *Salmonella* infection treated with AgNPs. The alleviation groups were orally administered (provided as water additives) with the AgNPs concentration at 5% w/v for 8 days, whereas the healthy group and *Salmonella* infection groups were orally administered with an equal volume of ddH_2_O. On the third day, the mice were gavaged with 0.2 ml of 5 × 10^6^ CFUs/ml *Salmonella* or an equivalent amount of phosphate‐buffered saline (PBS). The changes in body weight and mortality were recorded daily, and mice were killed on Day 5 post infection. The quantification of *Salmonella* in the fecal, colon, hepatic, spleen, and blood was measured per gram in all groups. Viable counts were determined as previously described^24^. The actual count was calculated using the following equation:

$$\text{CFU}/g=\left[10^{-(\text{dilution}\;\text{multiple})}\times \text{average}\;\text{number}\;\text{of}\;\text{colonies}\;\text{on}\;3\;\text{plates}\times 20\right]/\text{net}\;\text{sample}\;\text{weight}$$

In addition, colon length was measured, and liver, spleen, and colon tissues were also excised, sectioned into slices (5 µm), and observed via H&E staining.

### In vivo acute toxicity

Mice acclimated to their diet for a week were then split into six groups, including healthy ones and those treated with AgNPs. The toxicity groups were orally administered with formulations with the equivalent AgNPs concentration at 0.01, 0.1, 1, 10, and 100 mg/kg body weight for 1 week, whereas healthy control was orally administered with ddH_2_O. The changes in body weight and the probability of survival were recorded daily, and mice were killed on Day 10 after the administration of AgNPs. The liver, spleen, kidney, colon, duodenum, and ileum tissues were also excised, sectioned into slices (5 µm), and were observed via H&E staining.

### Statistical analysis

Data analysis and graphical representation were conducted using GraphPad Prism 9. The results were presented as the arithmetic mean, accompanied by the standard error of the mean (SEM). Typically, the statistical comparison between groups was undertaken using a two‐tailed Student's *t*‐test for dependent samples, whereas the comparison across several groups was executed via the one‐way analysis of variance (ANOVA) technique. A two‐way ANOVA was applied to assess variances across more than two groups with multiple factors, with the cell types and the treatment conditions serving as the variables of interest. Levels of significance were indicated as *p < 0.05, **p < 0.01, ***p < 0.001.

## AUTHOR CONTRIBUTIONS

**Xiuyan Jin**: Data curation (equal); formal analysis (equal); investigation (equal); methodology (equal); validation (equal); visualization (equal); writing—original draft (equal); writing—review and editing (equal). **Na Peng**: Investigation (equal); methodology (equal); validation (equal); visualization (supporting); writing—original draft (supporting). **Aoran Cui**: Data curation (supporting); methodology (supporting); validation (equal). **Yue Liu**: Methodology (supporting); validation (supporting). **Xianqi Peng**: Validation (supporting). **Linlin Huang**: Methodology (supporting); writing—original draft (supporting). **Abdelaziz Ed‐Dra**: Writing—review and editing (supporting). **Fang He**: Writing—review and editing (supporting). **Yan Li**: Supervision (supporting); validation (supporting); writing—review and editing (supporting). **Shikuan Yang**: Conceptualization (equal); resources (equal); writing—review and editing (equal). **Min Yue**: Conceptualization (equal); data curation (equal); formal analysis (equal); funding acquisition (equal); investigation (equal); methodology (equal); project administration (equal); resources (equal); software (equal); supervision (equal); validation (equal); visualization (equal); writing—original draft (equal); writing—review and editing (equal).

## ETHICS STATEMENT

Female C57BL/6 mice (6–8 weeks) were purchased from SLAC laboratory animals (Shanghai, China). Animals had free access to rat chow and tap water. All animal experiments were conducted according to the Guide for the Care and Use of Laboratory Animals and approved by the committee of Zhejiang University (Ethical Approval ZJU20220193).

## CONFLICT OF INTERESTS

The authors declare no conflict of interests.

## Supporting information

Supporting information.

## Data Availability

The data and supporting information are included in this article.
