# Supplementary material for: Sodium dodecyl sulfate‐coated silver nanoparticles accelerate antimicrobial potentials by targeting amphiphilic membranes
Source: mLife. 2024 Dec 3;3(4):551–64. doi: 10.1002/mlf2.12143 (PMC11686091; doi:10.1002/mlf2.12143)
Supplement: Supplementary file 1 — Supporting information. [file MLF2-3-551-s001.docx]

**Supporting Information**

**Sodium Dodecyl Sulfate-coated silver nanoparticles accelerate antimicrobial potentials by targeting amphiphilic membranes**

Xiuyan Jin ^a, 1^, Na Peng ^a 1^, Aoran Cui ^b^, Yue Liu ^b^, Xianqi Peng ^a^, Linlin Huang ^a^, Abdelaziz Ed-Dra ^c^, Fang He ^a, d^, Yan Li ^a, d, e^, Shikuan Yang ^b *^, Min Yue ^a, f, g *^

^a^ Department of Veterinary Medicine, Zhejiang University College of Animal Science, Hangzhou, China

^b^ Institute for Composites Science Innovation, School of Materials Science and Engineering, Zhejiang University, Hangzhou, China

^c^ Laboratory of Engineering and Applied Technologies, Higher School of Technology, M’ghila University Campus, Sultan Moulay Slimane University, BP: 591, Beni Mellal, Morocco

^d^ Laboratory of Animal Virology of Ministry of Agriculture, Zhejiang University Hangzhou, China

^e^ Hainan Institute, Zhejiang University, Sanya, China

^f^ State Key Laboratory for Diagnosis and Treatment of Infectious Diseases, National Clinical Research Center for Infectious Diseases, National Medical Center for Infectious Diseases, The First Affiliated Hospital, College of Medicine, Zhejiang University, Hangzhou, China

^g^ Key Laboratory of Systems Health Science of Zhejiang Province, School of Life Science, Hangzhou Institute for Advanced Study, University of Chinese Academy of Sciences, Hangzhou, China

^1^ Xiuyan Jin and Na Peng contributed equally to this work

* Corresponding authors: Min Yue ([myue@zju.edu.cn](mailto:myue@zju.edu.cn)); Shikuan Yang ([shkyang@zju.edu.cn](mailto:shkyang@zju.edu.cn))

E-mail addresses:

XJ (22017052@zju.edu.cn)

NP (pengna06@163.com)

YL (412531141@qq.com)

AC (12126053@zju.edu.cn)

XP (11817035@zju.edu.cn)

LH (22217045@zju.edu.cn)

AE-D (Abdelaziz_iaa@yahoo.fr); ORCID ID (0000-0003-3273-1767)

FH (hefangzj@zju.edu.cn)

YL ([yanli3@zju.edu.cn](mailto:yanli3@zju.edu.cn))

This file includes: Supplemental material and methods

Supplemental figure and figure legends

Supplemental table

# Supplemental Materials and Methods

**Biofilm inhibition assay**

The biofilm inhibition assay determined the potential of AgNPs and AgNO_3_ to inhibit initial bacterial cell adhesion. The protocol was previously documented^1^. The bacterial suspension was transferred into a 96-well plate. After incubation for 6 h, AgNPs or AgNO_3_ were added to the wells for 100 µL/well (final concentration is 5 mg/mL). Sterile LB broth was added to the control group and further incubated for 24 h at 22°C and 37°C in a static condition. Afterwards, 0.4 % crystal violet was added to each well. The assay was conducted in triplicate, and the percentage of biofilm inhibition was calculated using the following formula:

Biofilm inhibition (%) = [(OD _control_ – OD _treatment_)/OD _control_] × 100%

**Effect of AgNPs on the kinetic of bacterial growth**

A time-dependent killing curve analysis was conducted to evaluate the bactericidal activity of AgNPs and AgNO_3_ on four standard strains (*S. aureus, S.* Typhimurium, *L. monocytogenes,* and *E. coli*) for 48 h^2^. It was carried out in AgNPs or AgNO_3_ with a final concentration of 1 × MIC, 2 × MIC, 4 × MIC (MIC is 78.125 µg/mL of AgNPs for all examined bacteria), and 3 × 10^9^ CFU/mL bacteria. The untreated bacteria were used as the control.

# References

1. Paudyal N, Pan H, Elbediwi M, Zhou X, Peng X, Li X, et al. Characterization of *Salmonella* Dublin isolated from bovine and human hosts. *BMC Microbiol*. 2019;19:226.

2 . Mohd Yusof H, Abdul Rahman N, Mohamad R, Hasanah Zaidan U, Samsudin A. A. Antibacterial potential of biosynthesized zinc oxide nanoparticles against poultry-associated foodborne pathogens: an in vitro study. *Animals*. 2021;11, 2093.

# Supplemental Figures and Figure Legends


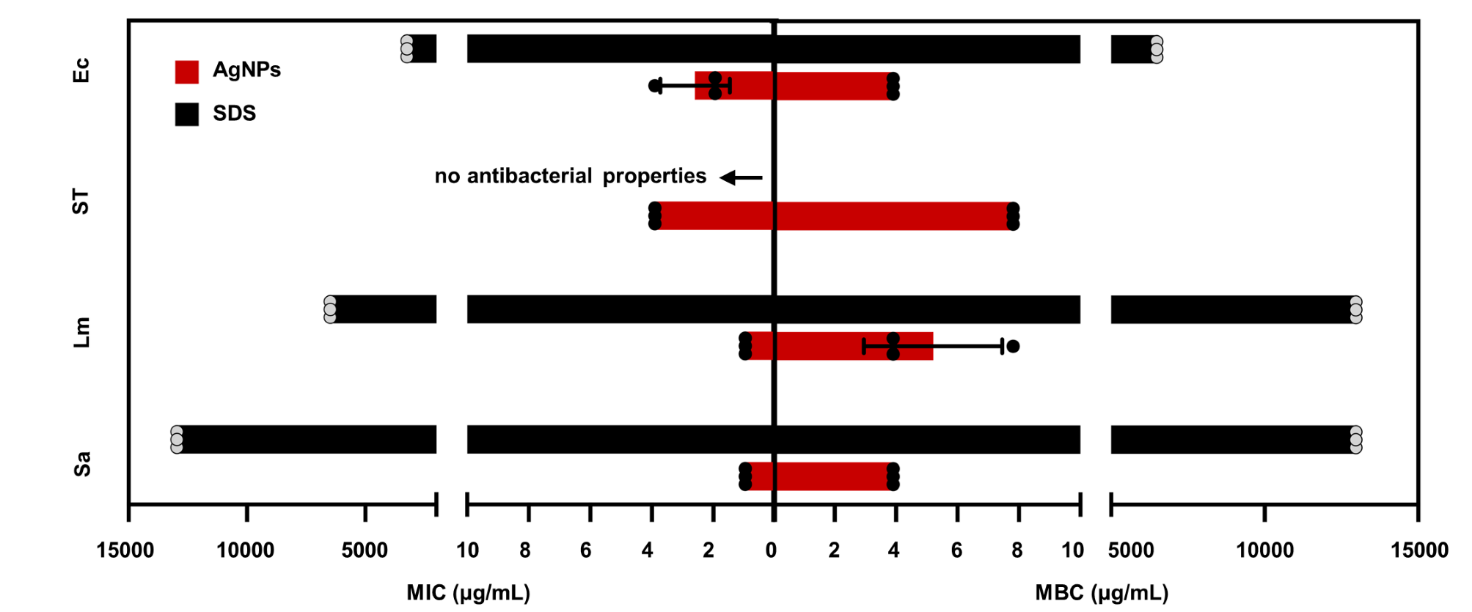


**Supplementary Figure 1. The bactericidal effect of AgNPs and pure SDS.** The minimum inhibitory concentration (MIC) and minimum bactericidal concentration (MBC) of AgNPs and pure sodium dodecyl sulfate (SDS). The dodecyl sulfate ligands without binding to the Ag nanoparticles have fragile antibacterial activity. Red: bacteria treated with AgNPs. Black: bacteria treated with pure SDS. Data were from at least three independent assays: Sa: *S. aureus*; Lm: *L. monocytogenes*; ST: *S.* Typhimurium; Ec: *E. coli*.


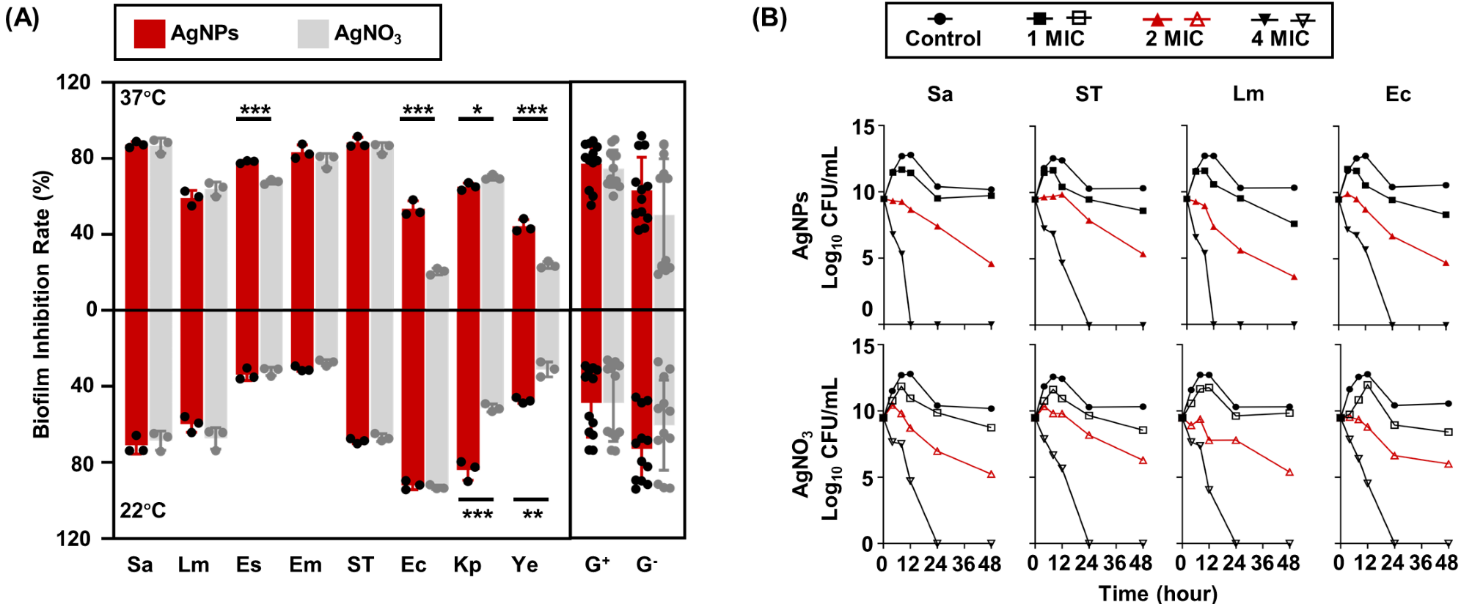


**Supplementary Figure 2. The bactericidal effect of AgNPs *in vitro*.** (**A**) Inhibition rate for bacterial biofilm formation at 37 ℃ and 22 ℃. (**B**) The growth curve of bacteria (log_10_ CFU/mL) treated with AgNPs and AgNO_3_ 1 ×, 2 ×, and 4 × the minimal inhibitory concentration (MIC) at different time intervals (0, 12, 24, 36, and 48 h). * *p* < 0.05; ** *p* < 0.01; *** *p* < 0.001. Sa: *S. aureus*; Lm: *L. monocytogenes*; Es: *E. faecalis*; Em: *E. faecium*; ST: *S.* Typhimurium; Ec: *E. coli*; Kp: *K. pneumoniae*; Ye: *Y. enterocolitica*.


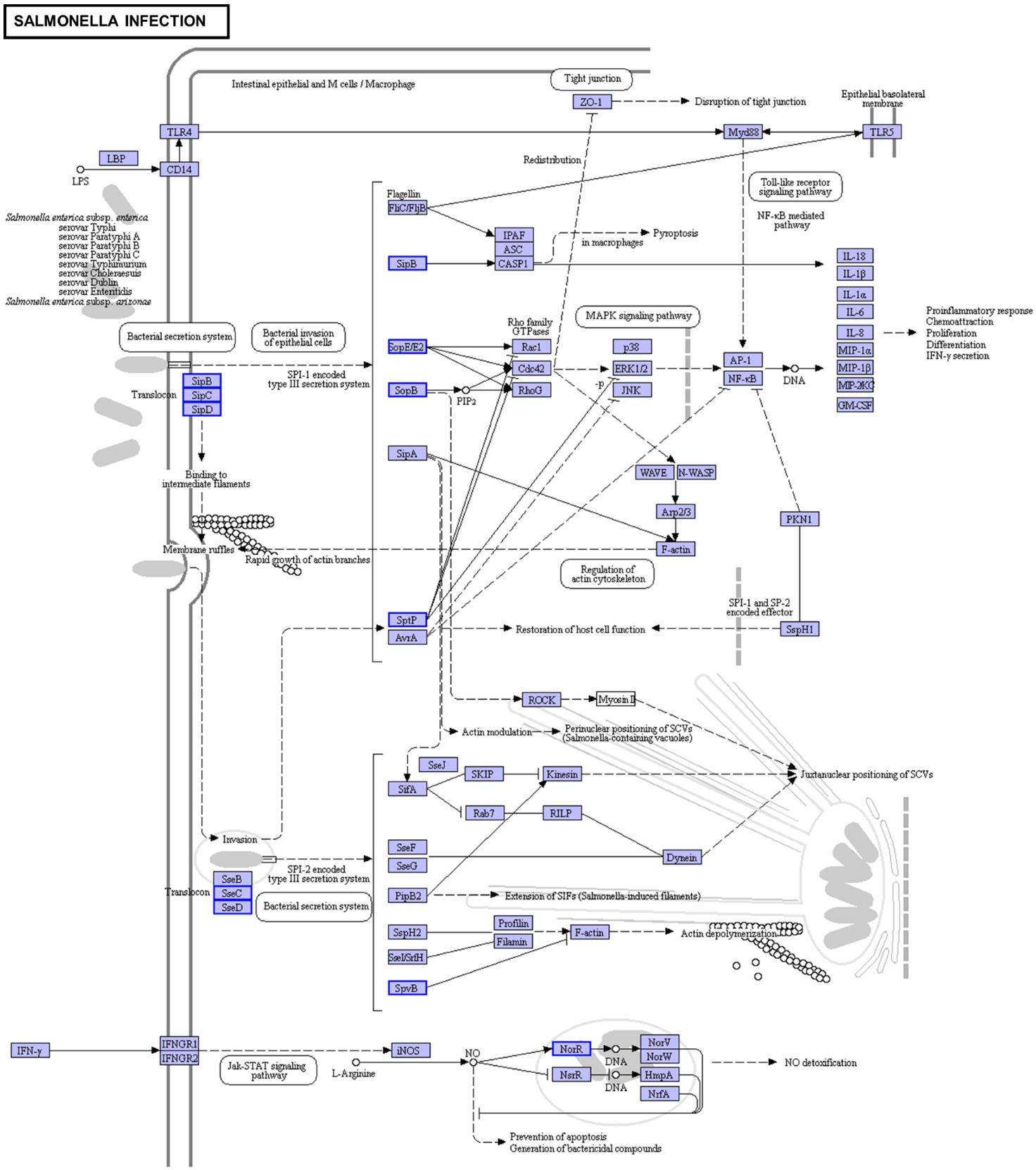


**Supplementary Figure 3. KEGG graph for *Salmonella* infection**


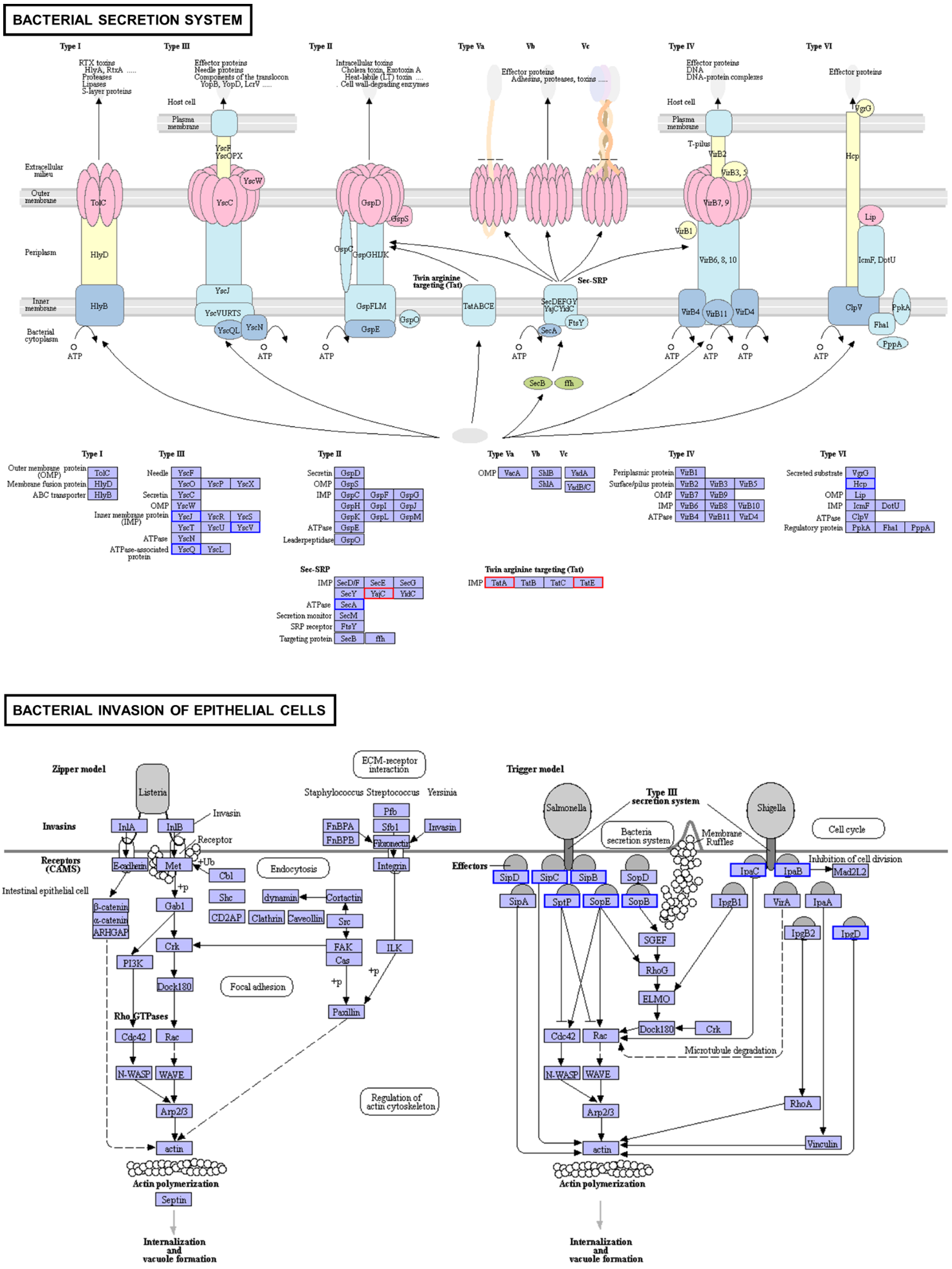


**Supplementary Figure 4. KEGG graphs for bacterial secretion system and bacterial invasion of epithelial cells**

# Supplemental Table

**Supplemental Table 1**. Metabolic pathways involved in differentially expressed genes.

| **Gene ID** | **Gene Name** | **Ratio** | **Description** |
| --- | --- | --- | --- |
| **Glycerophospholipid metabolism** |  |  |  |
| gene-SL1344_RS02150 | SL1344_RS17900 | -1.2 | aspartate aminotransferase family protein |
| gene-SL1344_RS11715 | *argC* | -1.4 | N-acetyl-gamma-glutamyl-phosphate reductase |
| gene-SL1344_RS11730 | *argG* | -1.4 | argininosuccinate synthase |
| gene-SL1344_RS11735 | *argA* | -1.1 | amino-acid N-acetyltransferase |
|  |  |  |  |
| **Fatty acid degradation** |  |  |  |
| gene-SL1344_RS12250 | *fadJ* | 1.3 | fatty acid oxidation complex subunit alpha FadJ |
| gene-SL1344_RS12255 | *fadI* | 1.1 | acetyl-CoA C-acyltransferase FadI |
|  |  |  |  |
| **Arginine biosynthesis** |  |  |  |
| gene-SL1344_RS22905 | *argF* | -2.8 | ornithine carbamoyltransferase |
| gene-SL1344_RS22915 | *arcA* | -2.0 | arginine deiminase |
| gene-SL1344_RS22910 | *arcC* | -1.9 | carbamate kinase |
| gene-SL1344_RS21180 | *argH* | -1.7 | argininosuccinate lyase |
| gene-SL1344_RS21175 | *argB* | -1.4 | acetylglutamate kinase |
| gene-SL1344_RS21170 | *argC* | -1.4 | N-acetyl-gamma-glutamyl-phosphate reductase |
| gene-SL1344_RS16990 | *argG* | -1.4 | argininosuccinate synthase |
| gene-SL1344_RS17900 | SL1344_RS17900 | -1.2 | aspartate aminotransferase family protein |
| gene-SL1344_RS15475 | *argA* | -1.1 | amino-acid N-acetyltransferase |
|  |  |  |  |
| **Oxidative phosphorylation** |  |  |  |
| gene-SL1344_RS02250 | SL1344_RS02250 | -2.2 | cytochrome o ubiquinol oxidase subunit IV |
| gene-SL1344_RS11935 | *nuoC* | -1.3 | NADH-quinone oxidoreductase subunit C/D |
| gene-SL1344_RS22295 | *frdB* | -1.3 | fumarate reductase iron-sulfur protein |
| gene-SL1344_RS11940 | *nuoB* | -1.2 | NADH-quinone oxidoreductase subunit NuoB |
| gene-SL1344_RS11930 | *nuoE* | -1.2 | NADH-quinone oxidoreductase subunit NuoE |
| gene-SL1344_RS03745 | *cydA* | -1.1 | cytochrome ubiquinol oxidase subunit I |
| gene-SL1344_RS02255 | SL1344_RS02255 | -1.0 | cytochrome o ubiquinol oxidase subunit III |
|  |  |  |  |
| **RNA degradation** |  |  |  |
| gene-SL1344_RS04150 | *rhlE* | 1.4 | 、 |
| gene-SL1344_RS22390 | *hfq* | 1.3 | RNA chaperone Hfq |
|  |  |  |  |
| **Biotin metabolism** |  |  |  |
| gene-SL1344_RS04030 | *bioC* | -2.8 | malonyl-ACP O-methyltransferase BioC |
| gene-SL1344_RS04025 | *bioF* | -2.6 | 8-amino-7-oxononanoate synthase |
| gene-SL1344_RS04015 | *bioA* | -2.5 | adenosylmethionine--8-amino-7-oxononanoate transaminase |
| gene-SL1344_RS04020 | *bioB* | -2.4 | biotin synthase BioB |
| gene-SL1344_RS04035 | *bioD* | -1.2 | ATP-dependent dethiobiotin synthetase BioD |
|  |  |  |  |
| **Pyruvate metabolism** |  |  |  |
| gene-SL1344_RS03885 | *oadA* | -7.4 | sodium-extruding oxaloacetate decarboxylase subunit alpha |
| gene-SL1344_RS22295 | *frdB* | -1.3 | fumarate reductase iron-sulfur protein |
| gene-SL1344_RS04265 | SL1344_RS04265 | -1.1 | glycyl radical protein |
| gene-SL1344_RS21160 | *ppc* | -1.1 | phosphoenolpyruvate carboxylase |
| gene-SL1344_RS07300 | *fumC* | -1.1 | class II fumarate hydratase |
| gene-SL1344_RS11995 | *pta* | -1.1 | phosphate acetyltransferase |
|  |  |  |  |
| **Ribosome** |  |  |  |
| gene-SL1344_RS00220 | *rpsT* | 2.5 | 30S ribosomal protein S20 |
| gene-SL1344_RS21045 | *rpmE* | 2.2 | 50S ribosomal protein L31 |
| gene-SL1344_RS17275 | *rpsI* | 1.8 | 30S ribosomal protein S9 |
| gene-SL1344_RS11425 | *rplY* | 1.5 | 50S ribosomal protein L25 |
| gene-SL1344_RS17280 | *rplM* | 1.3 | 50S ribosomal protein L13 |
| gene-SL1344_RS17065 | *rpmA* | 1.3 | 50S ribosomal protein L27 |
| gene-SL1344_RS22540 | *rpsF* | 1.3 | 30S ribosomal protein S6 |
| gene-SL1344_RS21315 | *rplL* | 1.1 | 50S ribosomal protein L7/L12 |
| gene-SL1344_RS17725 | *rplP* | -1.1 | 50S ribosomal protein L16 |
| gene-SL1344_RS19205 | *rpmB* | 1.1 | 50S ribosomal protein L28 |
| gene-SL1344_RS22555 | *rplI* | 1.0 | 50S ribosomal protein L9 |

**Supplemental Table 2**. The DEPs in bacterial invasion of epithelial cells, *salmonella* infection and bacterial secretion system pathways

| **Gene ID** | **Gene Name** | **Ratio** | **Description** |
| --- | --- | --- | --- |
| **Bacterial invasion of epithelial cells** |  |  |  |
| gene-SL1344_RS14900 | *sptP* | -1.0 | SPI-1 type III secretion system effector GTPase-activating protein SptP |
| gene-SL1344_RS14925 | *sipD* | -1.2 | SPI-1 type III secretion system needle tip complex protein SipD |
| gene-SL1344_RS14930 | *sipC* | -2.2 | SPI-1 type III secretion system needle tip complex protein SipC |
| gene-SL1344_RS05360 | *sopB* | -2.3 | SPI-1 type III secretion system effector inositol phosphate phosphatase SopB |
| gene-SL1344_RS14935 | *sipB* | -2.4 | SPI-1 type III secretion system needle tip complex protein SipB |
| gene-SL1344_RS13925 | *sopE* | -2.4 | SPI-1 type III secretion system guanine nucleotide exchange factor SopE |
|  |  |  |  |
| ***Salmonella* infection** |  |  |  |
| gene-SL1344_RS14705 | *norR* | -1.0 | nitric oxide reductase transcriptional regulator NorR |
| gene-SL1344_RS14900 | *sptP* | -1.0 | SPI-1 type III secretion system effector GTPase-activating protein SptP |
| gene-SL1344_RS06955 | *sseC* | -1.2 | SPI-2 type III secretion system translocon protein SseC |
| gene-SL1344_RS14925 | *sipD* | -1.2 | SPI-1 type III secretion system needle tip complex protein SipD |
| gene-SL1344_RS24000 | *spvB* | -1.2 | SPI-2 type III secretion system effector NAD(+)--protein-arginine  ADP-ribosyltransferase SpvB |
| gene-SL1344_RS06960 | *sseD* | -1.5 | SPI-2 type III secretion system translocon protein SseD |
| gene-SL1344_RS14930 | *sipC* | -2.2 | SPI-1 type III secretion system needle tip complex protein SipC |
| gene-SL1344_RS05360 | *sopB* | -2.3 | SPI-1 type III secretion system effector inositol phosphate phosphatase SopB |
| gene-SL1344_RS14935 | *sipB* | -2.4 | SPI-1 type III secretion system needle tip complex protein SipB |
| gene-SL1344_RS13925 | *sopE* | -2.4 | SPI-1 type III secretion system guanine nucleotide exchange factor SopE |
|  |  |  |  |
| **Bacterial secretion system** |  |  |  |
| gene-SL1344_RS03190 | *tatE* | 1.6 | twin-arginine translocase subunit TatE |
| gene-SL1344_RS02080 | *yajC* | 1.5 | preprotein translocase subunit YajC |
| gene-SL1344_RS20425 | *tatA* | 1.3 | Sec-independent protein translocase subunit TatA |
| gene-SL1344_RS00690 | *secA* | -1.1 | preprotein translocase subunit SecA |
| gene-SL1344_RS07025 | *ssaV* | -1.1 | SPI-2 type III secretion system apparatus protein SsaV |
| gene-SL1344_RS01415 | SL1344_RS01415 | -1.8 | type VI secretion system tube protein Hcp |
| gene-SL1344_RS14965 | *spaO* | -1.8 | SPI-1 type III secretion system protein SpaO |
| gene-SL1344_RS07000 | *ssaJ* | -1.9 | SPI-2 type III secretion system apparatus lipoprotein SsaJ |
| gene-SL1344_RS07020 | *ssaM* | -2.4 | SPI-2 type III secretion system apparatus protein SsaM |

**Supplemental Table 3**. The primers for RT-qPCR

| **Gene Name** | **Primer sequence (5'→3')** | **Product length** |
| --- | --- | --- |
| SL1344_RS17305 | TCTGGCGTCAACTCAGCAAT | 134 |
|  | CCGATACCCTGAGCCCAATC |  |
| *bioB* | CGTTGCTGCTGCTCGTTATC | 164 |
|  | CAGGCGATGTGCTTTATGGC |  |
| *fadJ* | CCAGCGAACGCGATAAACAG | 190 |
|  | CCAATCGGCAGGGAAGAAGT |  |
| *zraP* | CCCTCTCTCTTTTGGCCCTG | 113 |
|  | CGCCTGCTGTTCCGTAGTTA |  |
| *ccmE* | CTATACCCCCGGCGAAATCC | 179 |
|  | CCTCATAGCTCACCGTCACC |  |
| *16S rRNA* | TGTAGCGGTGAAATGCGTAG | 161 |
|  | CAAGGGCACAACCTCCAAG |  |
